# The inheritance of light

**DOI:** 10.1017/S1478951526102703

**Published:** 2026-05-13

**Authors:** Tara Rajendran

**Affiliations:** Department of Music, Faculty of Fine Arts, Annamalai University, Chidambaram, Tamil Nadu, India

## 1993-1997

Biologists say I lived within her briefly –
as all maternal grandchildren do.
Yet unlike other grandmothers,
she never carried me on her waist.
Or held my tiny palm in hers.
But I do remember sleeping beside her
And, she told the *Aesop’s* tales,
Not many, just two, perhaps three.

Dark-lit room, more so in the monsoon.
Late-1990s *Kerala* without electricity.
Emerald mango branches and coconut fronds
towered over the tiled pitched roof.
Hospital records tucked into window lattices.
Odor of half-air-dried cotton clothes.
Of petrichor wafting from outside.
Of breath, sweat, and toilet pan.

The long red tape recorder-cum radio,
which wafted Classical Indian music inside.
*Ragas* melted into that not-so-cosy afternoon.
Both calmly drifted off to vivid siesta dreams.

## 1998

The bright, spacious top-floor patient room.
Summer sunlight flooded through the window,
as though the vault of the cobalt sky had opened.
I can still catch the room’s scent from 1998.
Of floor cleaners, fresh bedsheets, and arid air
Of my new green teddy, whose eye I operated on later.
——

She perched in a perfect corner of our backyard,
Where lilac gliricidia sheds blossoms in bunches.
Wild jasmine spread a soft fragrance.
Swarms of fireflies flashed at midnight.
A cone of light fell on her every evening,
Schools of midges circled the cone of light.
Thin echoes of Pied-bush chats’ conversations.
Hum of neighborhood children’s classical music lessons

## 1999

I inherited her handwritten letters from 1989.
An Indian brass pocket bell, her grandfather gifted,
Gold jewelry made for my mom.
A set of multicolored *bindi* powders.
Pairs of soft cotton *mundu.*
Tiny jar with shining decorative stones.
Eggshell-white chiffon *saree*, red bordered.
And, the red radio and her idea of classical music.

## 1999-present

Her sense of music lingered in me –
years folded into decades.
26 years later, at my concerts,
I search for her among people,
All I seek is a cone of light.
A cone of light.

